# “Overcoming the Fear That Haunts Your Success” – The Effectiveness of Interventions for Reducing the Impostor Phenomenon

**DOI:** 10.3389/fpsyg.2020.00405

**Published:** 2020-05-15

**Authors:** Mirjam Zanchetta, Sabine Junker, Anna-Maria Wolf, Eva Traut-Mattausch

**Affiliations:** Department of Psychology, Paris-Lodron-University of Salzburg (PLUS), Salzburg, Austria

**Keywords:** impostor phenomenon, mindset theory, intervention, coaching, training

## Abstract

The impostor phenomenon (IP) refers to intense thoughts of fraudulence reported by high-achieving individuals. Since it has been shown to account for several personal and work-related complications, effective interventions are greatly needed. Against the background of mindset theory, we developed and tested two mindset interventions. We evaluated the impact of a coaching and a training intervention adopting a randomized controlled outcome design. One hundred and three young employees were randomly assigned to receive coaching (*n* = 36), training (*n* = 33), or no intervention (*n* = 34). Results reveal that coaching was an effective mindset intervention for sustainably reducing IP scores. Fear of negative evaluation emerged to mediate the relation between the coaching intervention and the reduced IP scores significantly. Moreover, coaching improved self-enhancing attributions and self-efficacy and reduced the tendency to cover up errors as well as the fear of negative evaluation. Training was superior in regard to knowledge acquisition. Specific implications are discussed.

## Introduction

The impostor phenomenon (IP) refers to intense thoughts of intellectual and/or professional fraudulence despite verifiable achievements; it prevents high-achieving individuals from being proud of their success and exploiting their maximum potential (Clance and Imes, 1978; Neureiter and Traut-Mattausch, 2016a). People with high expressions of the IP believe that their success is due to some kind of luck or error, and they live in constant fear of being exposed as unintelligent or less competent (Clance, 1985; Harvey and Katz, 1985; Jöstl et al., 2012). In early research on the IP it was also called the impostor syndrome, as it was thought to be somehow pathological. Research along these lines focused on personal, health-relevant consequences, and studies found that it caused psychological distress, lower well-being, (social) anxiety, and depression (Chrisman et al., 1995; Henning et al., 1998; Thompson et al., 1998; September et al., 2001; Bernard et al., 2002; Oriel et al., 2004). More recently, the syndrome has been thought to be a set of non- or subclinical cognitive features (e.g., Vergauwe et al., 2015; Neureiter and Traut-Mattausch, 2016a), and the IP has emerged as a more suitable term. Diverse expressions of the IP have been recognized in different cultures (Chae et al., 1995; Clance et al., 1995) as well as in different groups, such as marketing managers (Fried-Buchalter, 1997), undergraduate entrepreneurs (Sightler and Wilson, 2001), engineering students (French et al., 2008), medical, dental, nursing, and pharmacy students (Henning et al., 1998), and residents in family medicine (Oriel et al., 2004) and internal medicine (Legassie et al., 2008). In accordance with the original description of the IP (Clance and Imes, 1978), IP thoughts develop based on an individual’s learning history, starting in childhood, in terms of developmental lessons of correlation and causality. People with high expressions of the IP make attributions that inhibit any growth in self-esteem (e.g., Cozzarelli and Major, 1990). In cases of success, they attribute it to factors other than ability, such as some kind of luck or charm or knowing the right people (Clance and O’Toole, 1987; Cozzarelli and Major, 1990). They do not internalize their achievements and remain fearful of failing the next time (see also “the impostor cycle”; Clance, 1985). Furthermore, individuals with high expressions of the IP have been shown to feel affectively worse and to suffer a greater loss in state self-esteem than those with low expressions of the IP if they subjectively failed in an exam (Cozzarelli and Major, 1990). Moreover, they overgeneralize the implications of single failure experiences for their global self-concept (Thompson et al., 1998).

Along the way, several—mostly negative—consequences regarding career- and work-related variables have been identified. For instance, the IP has been found to be negatively related to (research) self-efficacy beliefs (Jöstl et al., 2012; McDowell et al., 2015; Neureiter and Traut-Mattausch, 2017), organizational citizenship behavior, affective commitment, job satisfaction, and perceived organizational support (Grubb and McDowell, 2012; McDowell et al., 2015; Vergauwe et al., 2015; Neureiter and Traut-Mattausch, 2016b) in working people. Moreover, the IP has been shown to decrease career planning, career exploration, career striving, career decision making, and the motivation to lead (Neureiter and Traut-Mattausch, 2016a, 2017). Against this empirical background and recent published articles calling for interventions that may reduce the experience of IP thoughts (e.g., Neureiter and Traut-Mattausch, 2016a, 2017; Brauer and Proyer, 2017), we were inspired to create potential interventions that could be applied in the work context. Especially because, to the best of our knowledge, empirical examined interventions to reduce expressions of the IP totally lack.

### Theoretical Background

Dweck’s mindset theory (Dweck, 1986; Hong et al., 1999) offers a useful theoretical background for the career- and work-related consequences of the IP outlined above. It is especially appropriate as it deals with beliefs that influence responses to challenges and setbacks (Dweck and Leggett, 1988; Hong et al., 1999). This theory (for an overview see Burnette et al., 2013) posits that individuals believe human attributes are either fixed (entity theory; fixed mindset) or malleable (incremental theory; growth mindset). According to previous studies, such beliefs have serious implications for reactions to challenges and motivation and can affect whether one chooses to engage in or forgo demanding activities (Dweck and Leggett, 1988; Mueller and Dweck, 1998). For instance, entity theorists tend to fear failure feedback because they interpret it as evidence of their inadequate ability, whereas incremental theorists tend to be less fearful of such feedback because they consider it useful information that supports the longer term goals of learning and developing mastery (Burnette et al., 2013). Langford (1990) postulated a link between the IP and an entity approach (Dweck, 1986), stating that the IP is related to a cognitive mindset, in which attributes such as intelligence are viewed as stable traits and mistakes are believed to indicate personal failure and inadequacy. This assumption earned further support when an empirical study, investigating if the IP affects individuals’ mindsets, showed that impostors—individuals with high expressions of the IP—truly tend to be entity theorists (Kumar and Jagacinski, 2006), in that they are convinced that attributes such as abilities or intelligence cannot be changed and are represented as limited, stable entities. Impostors’ fixed mindsets will prevent them from experiencing growth in confidence in their ability after achieving success at work. Instead, their conviction of being less capable than others think they are remains stable, reinforcing their feelings of fraudulence – a so-called impostor cycle (see further Clance, 1985).

In the work context, if individuals affected by the IP have a fixed (entity) mindset, they will judge their ability to attain a given level of performance (self-efficacy; e.g., Bandura, 1977) as fixed, stable, and unchangeable. Under this assumption, their self-efficacy does not get the chance to grow through achieved accomplishments, as is the case for unaffected individuals who hold a growth mindset. Furthermore, the positive relation between the IP and the fear of failure (Ross et al., 2001; Kumar and Jagacinski, 2006; Neureiter and Traut-Mattausch, 2016a) is fairly clear from an entity mindset perspective. Impostors would tend to interpret failure as evidence of their constantly feared insufficient ability. This is also in line with some original assumptions regarding the IP that impostors overgeneralize failure and consider it internal and stable (Clance and Imes, 1978; Chae et al., 1995; Thompson et al., 1998). Findings that impostors show decreased levels of career planning, career exploration, career striving, career decision making, and motivation to lead (Neureiter and Traut-Mattausch, 2016a, 2017) are also plausible if it is assumed they do not believe in growth and might therefore tend to forgo demanding activities that would promote successful career development.

### Interventions for Reducing Expressions of the IP

This research suggests that interventions aimed at reducing the personnel consequences of the IP in the career and work context should focus on implementing a growth mindset (Yeager et al., 2016). Exhibiting a growth mindset has already been shown to reduce negative effects on academic achievement in vulnerable groups (Claro et al., 2016). From the perspective of cognitive neuroscience, a growth mindset induction contributes to better cognitive control (Schroder et al., 2014), which might be especially useful, given that impostors demonstrate an external locus of control and unstable, external attributions in successful achievement situations (e.g., Brauer and Wolf, 2016). Hence, potential interventions should focus first on participants’ understanding that their feelings and behaviors may stem from their way of thinking about ability and performance as stable and unchangeable (entity approach) and second on fostering a mindset from which development can take place (incremental approach). Participants should explore the idea of a growth mindset to generate alternative ways of thinking about their abilities. This could help improve impostors’ basic assumptions about the belief that their successful performance is due to some kind of luck (external-unstable-specific success attribution) or that mistakes indicate a personal deficiency (internal-stable-global failure attribution). We wanted to facilitate a growth mindset approach, where abilities are assumed to grow, where impostors’ failure and success attributions become more self-enhancing and their self-efficacy has a chance to increase. This means that individuals affected by the IP should learn to make internal-stable-global attributions in case of positive events (self-enhancing attributions) and no longer in case of negative ones (self-destructive attributions). As a result, IP-affected individuals should recognize their own competence and believe in the growth of abilities, even through learning from mistakes.

To create an appropriate intervention that fosters a mindset change, we considered that both a coaching and a training are thought to be helpful in reducing IP feelings as recommended by previous works (Clance, 1986; Klinkhammer and Saul-Soprun, 2009). We conducted an intervention study to evaluate the effectiveness of these intervention approaches.

### Intervention Study

To address the deeply rooted set of cognitive features associated with the IP, an intense intervention, in which participants are required to actively go through a deep reflection and introspection regarding their fixed mindset, might be most effective. According to the findings of Spence et al. (2008), interventions that rely more on methods of facilitation and support than on education are able to achieve greater health behavior change. This is where coaching comes to the fore. Coaching can be described as a goal-focused helping relationship (Grant and Cavanagh, 2004; Grant et al., 2010b) where a coach and a client engage in a collaborative effort to set personal goals and develop, monitor, evaluate, and modify goal-appropriate activities (Grant et al., 2009) tailored to the individual’s specific needs in the context of organizational-level goals (Grant, 2001; Bond and Seneque, 2013). Indeed, coaching has been found to be a useful tool for individual development and facilitation of positive experiences in the professional lives of non-clinical individuals (Grant, 2003; Grant et al., 2010b; Theeboom et al., 2014). Further support was provided by Theeboom et al. (2014) summary of their meta-analytic findings that “coaching is an effective tool for improving the functioning of individuals in organizations” (p. 12). In particular, empirical findings have indicated that it is probable that participants’ self-enhancing attributions and self-efficacy as well as their environmental mastery and self-acceptance are increased through coaching (Green et al., 2006; Finn, 2007; Spence and Grant, 2007; Spence et al., 2008; Moen and Skaalvik, 2009). Thus, it seems possible that a coaching intervention will increase some IP-related positive characteristics, thereby fostering a growth mindset. Moreover, applying coaching interventions has been shown to be effective in decreasing depression, anxiety, and stress (Grant, 2003, 2008; Green et al., 2007; Grant et al., 2009, 2010a), additional variables highly related to the IP (Chrisman et al., 1995; Thompson et al., 1998; Bernard et al., 2002; Oriel et al., 2004; McGregor et al., 2008). Furthermore, coaching could be an appropriate intervention for our purpose as it facilitates individuals’ cognitive reframing of work experiences and attitudes (Grant, 2001; Theeboom et al., 2014).

Altogether, coaching appears to have larger and more consistent positive effects on outcome criteria compared to other popular interventions in the organizational context (for an overview and recent meta-analytical findings see Theeboom et al., 2014; Sonesh et al., 2015; Jones et al., 2016). Accordingly, we created a coaching intervention aimed at an IP score reduction by fostering a growth mindset. As the IP involves a shame component, such an intervention must show high sensitivity. Forcing participants to reflect on their IP cognitions and the associated long-held fixed mindset may alienate them and produce resistors. We addressed this issue by labeling the intervention “Fit for the Job” and presenting it as one of a number of diverse resource-oriented further education offerings. We concentrated on susceptible individuals—young employees who still needed to establish themselves in their work environment (e.g., Clance and Imes, 1978). We attempted to work on their mindset by improving their attributional style (internal-stable-global attributions in case of positive events and no longer in case of negative ones) and their self-efficacy. In addition to increasing these beneficial mindset characteristics, applying a coaching intervention enabled us to work on IP-related negative features, such as impostors’ strong tendency to cover up errors and high fear of negative evaluation. Mindset theory suggests that by applying a coaching intervention, on the one hand, it might be possible to increase positive features related to a growth mindset and, on the other hand, decrease negative features related to a fixed mindset. Therefore, we considered working on two positive features, namely, self-enhancing attributions and self-efficacy, and on two negative ones, namely, tendency to cover up errors and fear of negative evaluation. Guided by mindset theory, we made use of these indicators of a growth and a fixed mindset. A mindset shift could have been induced by the intervention if a change in the scores of the presumed indicators had occurred.

As coaching is assumed to be especially effective when working on (positive) behavior change compared to interventions that rely on education (Spence et al., 2008), we created a group training as a control intervention. In the training, approximately the same issues were addressed, but the setting differed. Training in general can be seen as a planned and systematic process that promotes the acquisition of knowledge, skills, and attitudes through instruction, demonstration, and practice (e.g., Salas and Cannon-Bowers, 2001; Salas et al., 2012). In addition to this control intervention, we compared participants receiving our coaching (or training) intervention to participants who did not get any intervention during data collection, which enabled us to get particular information regarding the effects of the interventions.

Our first goal was to evaluate the interventions regarding their specific effects. In this respect, the specification of appropriate coaching and training outcome criteria was required. First of all, the acceptance of and specific need for interventions is an important issue for the implementation of learning and development activities in organizations (Kirkpatrick, 1994). Thus, our first outcome criteria are more basic level, referring to participants’ satisfaction and considering the intervention as beneficial and relevant. Since both interventions (coaching and training) provided support for the employees while they mastered their job role, we expected similar reactions in terms of participants’ satisfaction and utility judgments (Hypothesis 1).

Second, learning and development interventions are provided to build job-relevant knowledge, skills, or attitudes (Kirkpatrick, 1994). Considering the reported characteristic features of coaching and training, we assumed the training intervention would be superior to the coaching intervention in conveying content-related knowledge (Hypothesis 2). Conversely, we expected that coaching would be more beneficial for participants to acquire individualized strategies for successfully managing their career (Hypothesis 3).

Third, coaching is aimed at impacting organizational results through the achievement of clients’ personal coaching goals, and thus goal attainment is regarded as a key coaching outcome criterion (Jones et al., 2016). For instance, clients’ work related coaching goals could include analyzing and deliberately using personal strengths that facilitate the fulfillment of one’s job role, growing in confidence, or increasing performance. There is evidence that coaching effectively promotes the achievement of clients’ coaching goals (Theeboom et al., 2014) and that this impact goes beyond that of traditional development methods such as training (Losch et al., 2016). We therefore hypothesized that the coaching intervention would be superior in helping participants achieve their coaching goals (Hypothesis 4).

Fourth, the main objective of the interventions created was to facilitate a mindset shift from an entity to a growth mindset to reduce expressions of the IP. Building on previous research and emphasizing the positive impact of coaching on IP-related variables, we assumed that the coaching intervention would have the greatest power to reduce IP scores in comparison to the training intervention and no intervention condition (Hypothesis 5). Moreover, we intended to investigate if a fostered growth mindset is responsible for the assumed IP score reduction. Thus, we expected that reduced IP scores of participants in the coaching intervention condition would be demonstrated by increased growth mindset indicators, namely, self-enhancing attributions, and self-efficacy (Hypothesis 6a). With regards to reducing negative features associated with a fixed mindset, we expected that reduced IP scores of participants in the coaching intervention condition would be explained by a lower tendency to cover up errors and less fear of negative evaluation (Hypothesis 6b).

## Materials and Methods

### Design

We implemented a 3 × 3 factorial design with the between-subjects factor intervention condition (coaching intervention, training intervention, no intervention) and the within-subject factor time of measurement (preintervention [T0/T01], immediately after the intervention [T1], 5 weeks post-intervention [T2]).

### Participants and Procedure

In all, 70 male and 33 female participants with a mean age of *M* = 18.39 years (*SD* = 2.00) took part in all three waves of the survey. In terms of education, the largest proportion reported having a secondary school certificate (78%); the remaining participants reported having a higher school certificate (13%), a junior high school certificate (8%), a commercial school certificate (1%), or no school certificate (1%). Regarding their work experience, the largest percentage reported having 1–12 months (46%), followed by 13–24 months (37%), 25–36 months (16%), or more than 36 months (2%).

We recruited participants who were selected to be trainees in one of two companies that operate internationally in the industrial sector and are specialized in sensor technology in Germany. We used this sample as young employees were expected to be especially susceptible to IP cognitions. A total of 103 participants were randomly assigned to one of the three experimental conditions: 36 to the coaching intervention (35%), 33 to the training intervention (32%), and 34 to the no intervention condition (33%). The T0 questionnaire included demographic questions containing gender, age, nationality, education, and work experience as well as a scale for assessing IP scores and goal attainment. Moreover, we assessed motivation and the components of a potential analysis at this time point. Additionally, participants answered questions immediately after each session that have been used for another study purpose. Moreover, goal attainment was once again obtained immediately at the end of the first session (T01). The T1 questionnaire, administered immediately after the intervention, included questions about satisfaction and utility, career management, and the mediators attributional style, self-efficacy, tendency to cover up errors, and fear of negative evaluation. Furthermore, we again addressed IP scores and goal attainment at this measurement point and included a multiple choice questionnaire to assess content-related knowledge. The T2 questionnaire was administered 5 weeks after the end of the intervention and included measures assessing IP scores, goal attainment, tendency to cover up errors, fear of negative evaluation, and career management. All scales were presented online except for utility and satisfaction, which were assessed in paper-and-pencil form. In the end, participants were debriefed. As a reward, all participants could get the results of the potential analyses based on the evaluated variables of the Bochumer Inventory for work specific personality descriptions six factors BIP-6-F (Hossiep and Krügers, 2012).

As reported above, all interventions were labeled as resource-oriented further education offerings for optimizing job performance with the running title “Fit for the Job.” Each intervention consisted of nine 40-min units spread over three sessions (each 120 min) with 2-week intervals between the sessions.

#### Coaching Intervention

The coaching intervention took place in a one-on-one dyadic setting so that the client could work on his or her individual issues and goals with the personal coach. At the beginning, the coaches supported the participants in refining personal goals they had identified prior to the intervention into more well-defined outcome goals for the coaching process that met the criteria of goal-setting theory (e.g., SMART, Doran, 1981; Locke and Latham, 1990). The coaching intervention was characterized by systematic support in developing beliefs in a self-efficacious working self, corresponding to a growth mindset. Therefore, participants reflected on their abilities with the coach and were encouraged to ask for feedback from colleagues and other related parties regarding their strengths and development as a take-home exercise between sessions. The coach worked with the participants collaboratively on the feedback results during the coaching session to facilitate their beliefs in their performance. To foster a positive impression, participants were encouraged to write a “letter to myself” describing their individual findings and progress that the coach would send to them half a year after the coaching intervention ended. Moreover, participants reflected on their “inner drivers” and their “inner team” (Schulz von Thun, 2013). Another issue was the participants’ concern about mistakes (tendency to cover up errors) and their fear of negative evaluation. For instance, the participants received a newspaper article showing positive aspects of making mistakes and worked through it collaboratively with the coach. Furthermore, the coach and the participant reflected on the personal attributional style in the case of failure and success. The intervention ended with the evaluation of the coaching process and their goal attainment.

Coaches were master’s students in psychology who had successfully completed a professional supervised 1-year coaching training (about 220 h) with a focus on career planning (for education concept see Braumandl et al., 2013). In the first part of the training program, experienced coaches provided students with theoretical information and practical training on coaching-specific skills. These include, for example, questioning techniques to effectively facilitate clients’ goal attainment and self-reflection. Training exercises and the following peer-coaching sessions addressed issues relating to career planning, such as identification of strengths and potentials, specification of resources and competences, shaping values and meaning, and the development of action plans. The second part of the training program involved the practical application of coaching skills in client coaching, which was covered by three supervision sessions from professionally trained supervisors.

#### Training Intervention

As the control intervention was a training intervention, it was conducted in a group setting with 8–10 participants and one trainer. Given the nature of training and in aid of group processes, essential training elements such as ice breakers, introduction rounds, quick energizers, repetition of content to facilitate the transfer of the learning input, and feedback were implemented (Brinkmann, 2008; Döring, 2009; Weidemann, 2015). At the beginning, the trainer (as the coaches did) encouraged the participants to refine the personal goals they had identified prior to the intervention into more well-defined outcome goals that met the criteria of goal-setting theory (e.g., SMART, Doran, 1981; Locke and Latham, 1990). Therefore the participants had to write down concrete steps that needed to be taken to realize wishes that were expressed to a “training fairy” in advance. Also the training intervention was characterized by systematic support in developing beliefs of a self-efficacious working self, corresponding to a growth mindset, although it was based on theoretical concepts and group interaction. That is, participants reflected on their abilities and development with the people sitting next to them. The approach was based on theoretical input regarding social competence, interpersonal competence, and expertise. To foster a positive impression, participants were encouraged to make “paintings” of their individual findings and progress. Moreover, participants learned about the theory of “inner drivers” and the “inner team” (Schulz von Thun, 2013) and they discussed this in groups of three. As in the coachings, another issue was the participants’ concern about mistakes (tendency to cover up errors) and the fear of negative evaluation. In this intervention the participants received a newspaper article showing positive aspects of making mistakes and read through it with the other training participants. Furthermore, the trainer told participants about attributional style theory (Heider, 1958) and gave an example situation. The intervention ended with participants’ evaluation of the training process and their goal attainment.

The trainer was also a master’s student in psychology who had on the top of the coaching training described above successfully completed a professional train-the-trainer program. To ensure consistency in study procedure, we used guidelines to instruct the coaches and the trainer about the content and structure of each coaching/training session (for more details regarding the coaching and the training intervention, see Muck, 2015).

#### No Intervention Condition

Participants in this condition did not receive any intervention during the time of data collection but received a training intervention in time management afterward. The study design and procedure is displayed in Table 1.

**TABLE 1:** Overview of the study design and procedure.

Intervention sessions							

Condition	10 days		14 days			5 weeks	
			
	T0	Session 1	T01	Session 2	Session 3	T1	T2
Coaching intervention	x	x	x	x	x	x	x
Training intervention	x	x	x	x	x	x	x
No intervention	x		x			x	x

*Study procedure is depicted in chronological order from left to right; T0 = preintervention; T01 = immediately at the end of the first session; T1 = immediately after the intervention; T2 = post-intervention.*

### Measures

Unless noted otherwise, all items were rated on a five-point scale ranging from 1 (*not at all true*) to 5 (*very true*).

#### Motivation

To assess motivation, we used the external regulation subscale of the Situational Motivation Scale (SIMS; Guay et al., 2000). The scale consisted of four items (e.g., “Because I am supposed to do it”) and the introductory question was “Why are you attending the coaching/training/further education offering?” In the current sample, internal consistency was adequate (α_T__0_ = 0.77).

#### Satisfaction

To assess satisfaction, we used the eight-item scale by Mäthner et al. (2005). The scale was adapted for the coaching and the training intervention (e.g., “I was very satisfied with the coaching/training process”). The internal consistency in the current sample was adequate (α_T__1_ = 0.76).^1^

#### Utility

To assess the perceived utility of the intervention after it ended (T1), we used the two-item scale by Kauffeld et al. (2009). The first item was “How relevant has the intervention been for your job?” and the second item was “How beneficial was the intervention for future working tasks?” The items were rated on a scale ranging from 0 to 100% (in 10% increments). A correlation analysis revealed a significant correlation (*r*_T1_ = 0.38, *p* = 0.001).

#### Content-Related Knowledge

To assess the content-related knowledge conveyed by the interventions, we created a multiple choice questionnaire with 11 questions. Each question had four response options. Each correct answer earned 1 point. More points represent more content-related knowledge.

#### Career Management

To assess career management, we used the Career Management Scale developed by Gould (1979) and translated into German by Rowold (2004). The scale comprised nine items (e.g., “I know what to do to reach my career goals”). In the current sample, internal consistency was adequate to questionable (α_T__1_ = 0.73; α_T__2_ = 0.65).

#### IP Scores

We used the CIPS (Clance, 1985; Klinkhammer and Saul-Soprun, 2009; Brauer and Wolf, 2016) to measure the IP. We used it in a slightly modified version as we added an introductory sentence “In the last 2 weeks…” to assess the current IP occurrence. In the current sample, internal consistency for the CIPS was good (α_T__0_ = 0.87; α_T__1_ = 0.80; α_T__2_ = 0.86). For correlations across time points see Table 2.

**TABLE 2:** Correlations across time points regarding IP scores.

Condition	T0–T1		T1–T2		T0–T2	
			
	*r*	*p*	*r*	*p*	*R*	*p*
Coaching intervention	0.17	0.325	0.48*	0.003	0.29^†^	0.088
Training intervention	0.66*	<0.001	0.45*	0.009	0.69*	<0.001
No intervention	0.65*	<0.001	0.74*	<0.001	0.62*	<0.001

*N* = 103, ^†^*p* < 0.10, * *p* < 0.05.

#### Goal Attainment

The clarification of current issues and goal setting is one of the first activities in the establishment of a goal-focused coaching agreement. Clients might create goals before the intervention starts (T0), but evidence (Losch et al., 2016) suggests a more valid measure of goal attainment occurs when clients focus on specific goals after exploring them deeply in the first session (T01). Using T01 goal attainment scores as a baseline measure makes it possible to match baseline and post-intervention goal scores (T1, T2) more accurately. Therefore, participants rated their current degree of goal attainment at T0, T01, T1, and T2 by answering the question “As of right now, to what extent have you attained this goal?” (Braumandl and Dirscherl, 2005; Biberacher, 2010; Ianiro et al., 2014) on a scale ranging from 1 (*not at all achieved*) to 10 (*fully achieved*).

#### Attributional Style

To assess attributional style, from the attributional style questionnaire for adults ASF-E (Poppe et al., 2005) we used the 6 of 16 scenarios that were suitable for our participants. Three were positive events and three were negative events at work. An example for a positive event is: “The main reason that my supervisor gave me a compliment is….” And an example for a negative event is: “The main reason for being criticized on my job is…” The response options ranged from 1 (*lies within other people*) to 9 (*lies within me*) for internality; 1 (*will change over time*) to 9 (*will not change over time*) for stability; and 1 (*is only valid for this situation*) to 9 (*is valid for all situations*) for globality^2^. Internal-stable-global attributions in case of positive events were assigned to “self-enhancing attributions” and in case of negative events to “self-destructive attributions.” In the current sample, internal consistency was adequate for self-enhancing attributions (α_T1_ = 0.75) as well as for self-destructive attributions (α_T1_ = 0.71).

#### Self-Efficacy

To assess self-efficacy, we used the six-item scale (e.g., “When I am confronted with a problem in my job, I can usually find several solutions”) by Rigotti et al. (2008). In the current sample, internal consistency was adequate (α_T1_ = 0.75).

#### Tendency to Cover Up Errors

To assess the tendency to cover up errors, we used the six-item covering-up-errors subscale (e.g., “Why mention a mistake when it isn’t obvious?”) of the Error Orientation Questionnaire (Rybowiak et al., 1999; Bauer et al., 2004). The internal consistency was questionable (α_T__1_ = 0.61; α_T__2_ = 0.62) in the current sample. Nevertheless, we used it originally as an item reduction would not boost the internal consistency at all.

#### Fear of Negative Evaluation

To assess the fear of negative evaluation, we used the five-item Fear of Negative Evaluation Scale (e.g., “Sometimes, I think I am too concerned with what other people think of me”) by Kemper et al. (2012). In the current sample, internal consistency was good (α_T__1_ = 0.85; α_T__2_ = 0.80).

## Results

### Preliminary Analyses

Table 3 presents means and standard deviations of the study variables across intervention conditions and time of measurement. First, preintervention (T0/T01) differences across intervention conditions were analyzed using univariate ANOVAs. Significant main effects were followed by *post hoc* comparisons. The three groups were equivalent on IP scores at T0, *F*(2,100) = 0.04, *p* = 0.965, η^2^ = 0.00, but differed in goal attainment^3^ at T01, *F*(2,95) = 6.54, *p* = 0.002, η^2^ = 0.12. Pairwise comparisons revealed that goal attainment scores of the coaching intervention participants (*M*_T__01_ = 4.89, *SD* = 1.53, *p* = 0.043) and the no intervention participants (*M*_T__01_ = 5.57, *SD* = 2.00, *p* = 0.001) were significantly higher compared to those in the training intervention condition (*M*_T__01_ = 4.06, *SD* = 1.44). No further significant differences occurred (*p*s > 0.05). To examine the effect of the intervention conditions, post-intervention (T1) and 5 weeks post-intervention (T2) scores were subjected to ANCOVA with T0 IP and T01 goal attainment scores as the covariates.

**TABLE 3:** Means and standard deviations of the variables.

Variable	Condition					
	
	Coaching intervention		Training intervention		No intervention	
			
	*M*	*SD*	*M*	*SD*	*M*	*SD*
**T0**						
Motivation	3.35	1.11	2.91	0.82	3.18	1.13
IP scores	2.25	0.57	2.27	0.55	2.23	0.58
Goal attainment^a^	4.49	1.82	4.27	1.74	5.23	1.83
**T01**						
Goal attainment^a^	4.89	1.53	4.06	1.44	5.57	2.00
**T1**						
Satisfaction	3.72	0.46	3.69	0.41	–	–
Utility^b^	66.94	15.78	64.85	12.90	–	–
Content-related knowledge^c^	5.67	2.44	7.00	2.83	5.00	2.70
Career management	3.64	0.47	3.22	0.66	3.18	0.69
IP scores	1.95	0.28	2.11	0.33	2.36	0.54
Goal attainment^a^	6.60	2.08	5.45	1.68	5.77	2.16
**Mediating variables**						
Self-enhancing attributions	6.90	0.95	6.43	1.19	5.98	0.96
Self-destructive attributions	5.11	1.20	5.29	0.71	5.34	1.31
Self-efficacy	4.39	0.68	3.74	0.69	3.41	0.68
Tendency to cover up errors	1.87	0.61	2.19	0.60	2.24	0.56
Fear of negative evaluation	2.04	0.68	2.24	0.88	2.54	0.96
**T2**						
IP scores	1.79	0.30	2.12	0.48	2.22	0.52
Goal attainment^a^	6.66	2.36	5.58	1.52	5.87	1.93
Career management	3.49	0.61	3.25	0.48	3.15	0.57
Tendency to cover up errors	1.74	0.56	2.27	0.49	2.28	0.36
Fear of negative evaluation	1.98	0.63	2.23	0.67	2.59	0.66

**N* = 103. ^*a*^*N* = 98. Four participants were excluded from the analysis because they did not set a goal; one was excluded because of a missing value. ^*b*^Scores ranged from 0 to 100. ^*c*^Scores ranged from 0 to 12.*

We further evaluated the impact of participants’ motivation prior to the intervention and found that all participants in all three conditions (coaching intervention, training intervention, and no intervention) were equally motivated at T0, *F*(2,100) = 1.61, *p* = 0.204, η^2^ = 0.03. As motivation was not significantly associated with the outcome measures (over all conditions: all *r* < |0.18|, all *p*s > 0.05, separately for each condition: all *r* < |0.27|, all *p*s > 0.05), we did not control for this variable in the main analyses.

### Effectiveness of Interventions

#### Satisfaction and Utility Judgments (Hypothesis 1)

All participants of the coaching intervention and training intervention were equally satisfied, *F*(1,67) = 0.10, *p* = 0.750, η^2^ = 0.00, and made equal utility judgments about the intervention, *F*(1,67) = 0.36, *p* = 0.550, η^2^ = 0.01, after the intervention ended (T1). The results support Hypothesis 1.

#### Content-Related Knowledge (Hypothesis 2)

Participants in the training intervention condition scored significantly higher on the multiple choice test measuring content-related knowledge, *F*(2,100) = 4.93, *p* = 0.009, η^2^ = 0.09, compared to participants in the coaching intervention (*p* = 0.040, *d* = −0.51^4^) and the no intervention (*p* = 0.003, *d* = −0.72) condition. As further indicated by the LSD *post hoc* test, there was no difference between participants in the coaching intervention and the no intervention condition (*p* = 0.296, *d* = −0.26). Hence, the training intervention conveyed significantly more content-related knowledge, thereby supporting Hypothesis 2.

#### Career Management (Hypothesis 3)

Regarding the effects on career management, we conducted a 3 (Intervention Condition) × 2 (Measurement Time Point) ANOVA. As expected, participants in the coaching intervention condition showed significantly higher scores, *F*(2,100) = 6.10, *p* = 0.003, η^2^ = 0.11, compared to participants in the training intervention (*p* = 0.006, *d* = −0.74) and the no intervention (*p* = 0.002, *d* = −0.78) condition at T1. However, there was no difference between participants in the training intervention and the no intervention condition at T1 (*p* = 0.776, *d* = −0.06). A similar effect was less strong but still visible 5 weeks after the intervention (T2), *F*(2,100) = 3.52, *p* = 0.033, η^2^ = 0.07. Participants in the coaching intervention showed by tendency but not significant higher career management compared to participants in the training intervention (*p* = 0.070, *d* = −0.45) and significantly higher compared to the no intervention (*p* = 0.012, *d* = −0.58) condition. Again, there was no difference between participants in the training intervention and the no intervention condition at T2 (*p* = 0.483, *d* = −0.18). Neither the measurement time point effects nor the interaction effect reached statistical significance (both *F*s < 1, *p*s > 0.05). In sum, coaching intervention participants showed significantly higher career management that remained constant over time, lending support to Hypothesis 3.

#### Goal Attainment (Hypothesis 4)

Regarding goal attainment scores, we found a significant difference between the intervention conditions in the change in goal attainment at T1 when covarying the T01 scores, *F*(2,94) = 3.30, *p* = 0.041, η^2^ = 0.07. Pairwise comparisons revealed that goal attainment was significantly higher for coaching intervention participants compared to those in the no intervention condition, *p* = 0.015, *d* = −0.39, and by tendency but not significant higher compared to those in the training intervention, *p* = 0.095, *d* = −0.61. When comparing the goal attainment scores of participants in the training intervention with those of participants in the no intervention condition, no significant difference occurred, *p* = 0.446, *d* = 0.17. At the 5-week post-intervention assessment (T2), there was a difference in the change in goal attainment across groups, *F*(2,94) = 2.72, *p* = 0.071, η^2^ = 0.06. As indicated by pairwise comparisons, goal attainment scores of the coaching intervention participants were significantly higher than those of participants who did not receive any intervention, *p* = 0.031, *d* = −0.36. This time, participants in the training intervention did not differ significantly compared to those in the coaching intervention, *p* = 0.101, *d* = 0.54, or the no intervention condition, *p* = 0.599, *d* = 0.17. Again, subsequent ANOVAs and pairwise comparisons were performed to evaluate within-condition effects across time. No considerable changes in goal attainment were observed in the no intervention condition from T01 to T2, *p*s > 0.05, *d* = 0.16. By contrast, goal attainment scores of participants who received the coaching or training intervention increased significantly immediately after the intervention from T01 to T1, *p* < 0.001, *d* = 0.91, and *p* < 0.001, *d* = 0.81, respectively, and were maintained for 5 weeks after the intervention ended (*p* = 0.870, *d* = 0.03, and *p* = 0.735, *d* = 0.07, respectively). Overall, despite an observed large within-condition change in training intervention participants’ goal attainment scores, no significant differences compared to no intervention participants occurred at T1 and T2. Hence, we found support for our Hypothesis 4.

#### IP Scores (Hypothesis 5)

The IP scores were subjected to ANCOVA with preintervention scores (T0) as the covariate. There was a significant difference between the intervention conditions in the change in IP scores immediately after the intervention (T1), *F*(2,99) = 12.54, *p* < 0.001, η^2^ = 0.20. Pairwise comparisons revealed that the IP scores of participants in the coaching intervention, *p* < 0.001, *d* = 0.95, and training intervention, *p* = 0.003, *d* = 0.54, were lower than the IP scores of the participants who had no intervention. Participants who received the coaching tended to have lower IP scores than those who received the training intervention, *p* = 0.068, *d* = 0.52. Five weeks after the intervention ended (T2), the intervention conditions also differed in the change in IP scores, *F*(2,99) = 13.07, *p* < 0.001, η^2^ = 0.21. Pairwise comparisons indicated that participants who received coaching exhibited significantly lower IP scores than participants in the training intervention, *p* = 0.001, *d* = 0.80, and no intervention, *p* < 0.001, *d* = 1.02, condition. Comparing the IP scores of the training intervention with those of the no intervention participants, no significant difference occurred this time, *p* = 0.181, *d* = 0.21.

**FIGURE 1:**
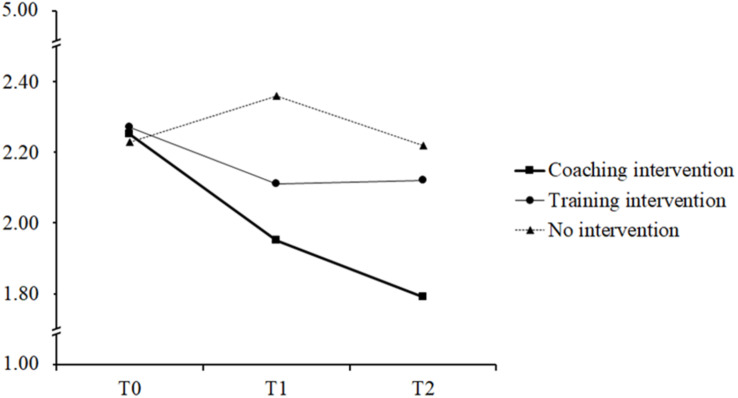
Imposter phenomenon scores by condition and measurement time point. T0 = preintervention; T1 = immediately after the intervention; T2 = 5 weeks post-intervention.

Subsequent ANOVAs and pairwise comparisons were used to reveal effects within each individual intervention condition across time. According to the reported means depicted in Table 3, IP scores significantly decreased in the coaching intervention, both from T0 to T1, *p* = 0.001, *d* = −0.41, and from T1 to T2, *p* = 0.012, *d* = −0.57, indicating a continuous decrease over time. IP scores of the training intervention participants, however, tended to decrease from T0 to T1, *p* = 0.077, *d* = −0.34, but not from T1 to T2, *p* = 0.982, *d* = 0.00 pointing to a slight reduction immediately after the intervention that remained stable for 5 weeks post-intervention. Participants in the no intervention condition showed no appreciable change in IP scores from T0 to T2, *p* = 0.889, *d* = −0.02. In sum, the results support our Hypothesis 5. Results are displayed in Figure 1.

### Mediation Analysis (Hypotheses 6a and 6b)

To examine which cognitions mediated the effects of the coaching intervention on IP scores (Hypotheses 6a and 6b), we conducted a parallel mediation analysis using the PROCESS macro offered by Hayes (2013). We calculated specific indirect effects using 5,000 bootstrap iterations and made use of Model 4. If the bias-corrected 95% confidence interval (95% BC CI) does not include zero, the indirect effect is considered to be significant. Our mediation analysis included attributional style, self-efficacy, tendency to cover up errors, and fear of negative evaluation. We employed Contrast A (coaching intervention vs. no intervention condition, training intervention vs. no intervention condition as covariate) as the independent variable to examine the effects on the dependent variable T2 IP scores.

**FIGURE 2:**
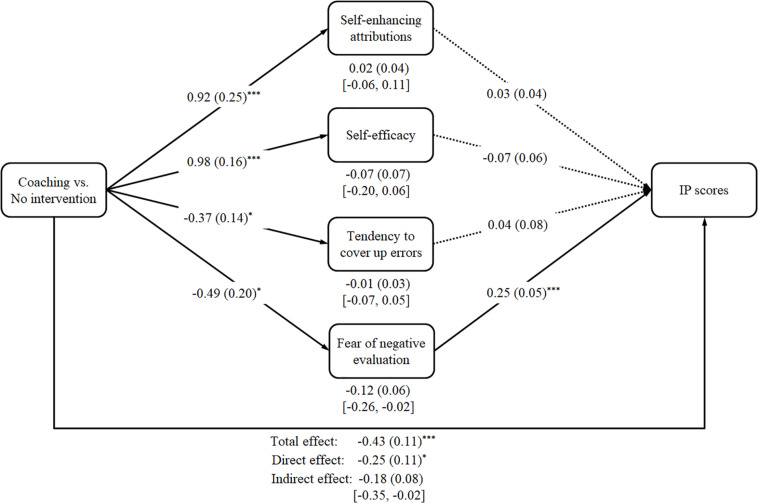
The effects of the coaching intervention (Contrast A) on IP scores via positive and negative features. Values in square brackets represent the 95% bias-corrected confidence interval for indirect effects. ^∗^*p* < 0.05; ^∗∗^*p* < 0.01; ^∗∗∗^*p* < 0.001.

The analysis revealed a significant total effect, *b* = −0.43, *SE* = 0.11, *t*(100) = −4.06, *p* < 0.001. When taking all mediators into account simultaneously, the prediction of T2 IP scores was less strong, *b* = −0.25, *SE* = 0.11, *t*(96) = −2.26, *p* = 0.026, but still significant. The coaching intervention (Contrast A) had a significant effect on self-enhancing attributions, *b* = 0.92, *SE* = 0.25, *t*(100) = 3.73, *p* < 0.001, on self-efficacy, *b* = 0.98, *SE* = 0.16, *t*(100) = 6.03, *p* < 0.001, on the tendency to cover up errors, *b* = −0.37, *SE* = 0.14, *t*(100) = −2.62, *p* = 0.010, as well as on the fear of negative evaluation, *b* = −0.49, *SE* = 0.20, *t*(100) = −2.43, *p* = 0.017. With regard to indirect effects, neither self-enhancing attributions, *b* = 0.02, *SE* = 0.04, 95% BC CI [−0.06, 0.11], nor self-efficacy, *b* = −0.07, *SE* = 0.07, 95% BC CI [−0.20, 0.06], nor the tendency to cover up errors, *b* = −0.01, *SE* = 0.03, 95% BC CI [−0.07, 0.05], had a significant indirect effect. However, we found a significant total indirect effect, *b* = −0.18, *SE* = 0.08, 95% BC CI [−0.35, −0.02], and a significant indirect effect regarding fear of negative evaluation, *b* = −0.12, *SE* = 0.06, 95% BC CI [−0.26, −0.02]. Analogously, only fear of negative evaluation had a significant effect on T2 IP scores, *b* = 0.25, *SE* = 0.05, *t*(96) = 4.98, *p* < 0.001, whereas self-enhancing attributions, *b* = 0.03, *SE* = 0.04, *t*(96) = 0.58, *p* = 0.564, self-efficacy, *b* = −0.07, *SE* = 0.06, *t*(96) = −1.17, *p* = 0.243, and the tendency to cover up errors, *b* = 0.04, *SE* = 0.08, *t*(96) = 0.47, *p* = 0.642, had no significant effect on T2 IP scores. Hence, we found no support for our Hypothesis 6a. However, there is some support for our Hypothesis 6b as the fear of negative evaluation emerged as significant mediating variable between our coaching intervention and reduced T2 IP scores. The mediation analysis is illustrated in Figure 2.

## Discussion

Based on previous research regarding the IP and mindset theory, the present investigation explored the impact of interventions to reduce IP scores. We compared a coaching intervention with a training intervention and no intervention. Evaluation analyses revealed that participants in the intervention conditions were equally satisfied after the coaching and training had ended. Moreover, all intervention participants made equal utility judgments about the interventions (supporting Hypothesis 1). Furthermore, participants in the training intervention obtained significantly higher knowledge scores compared to those who received the coaching or no intervention. This supports the considerations that led to Hypothesis 2, that training in general promotes the acquisition of knowledge (e.g., Salas and Cannon-Bowers, 2001; Salas et al., 2012) and may be superior in this regard. Moreover, coaching intervention participants showed significantly higher career management immediately as well as 5 weeks after the intervention, supporting Hypothesis 3. In line with our predictions concerning the achievement of personal coaching goals (Hypothesis 4), coaching resulted in a considerable increase in goal attainment that was sustained over time. This finding is consistent with research documenting that coaching is more beneficial than training to facilitate the achievement of personal goals (Losch et al., 2016). Overall, our research contributes to the recognition of the direction of clients’ goal-relevant efforts as a distinguishing characteristic of coaching conversations (Grant and Stober, 2006; Grant, 2012). We turn next to our main focus, participants’ IP scores. Starting with equal IP scores across all participants, the coaching intervention turned out to be superior, revealing a significantly higher reduction of participants’ IP scores in comparison to both the training and no intervention, thereby supporting Hypothesis 5. Since we found an effect of time in addition to an effect of condition, we can further conclude that the desired coaching intervention effect was not only sustainable, but even increased over time, according to the reported values. As stated in Hypotheses 6a and 6b, we investigated the coaching intervention’s effect on positive as well as negative features related to mindset theory and the IP. As expected, by applying a parallel mediation analysis we found that the coaching intervention was able to foster an increase in participants’ self-enhancing attributions and self-efficacy, as reported previously (Green et al., 2006; Finn, 2007; Spence and Grant, 2007; Spence et al., 2008; Moen and Skaalvik, 2009). However, the increased self-efficacy was not significantly related to decreased IP scores, as predicted by previous research (Jöstl et al., 2012; McDowell et al., 2015; Neureiter and Traut-Mattausch, 2017). The findings on self-enhancing attributions were also less clear, as increased self-enhancing attributions did not cause decreased IP scores as well. These results require further discussion as well as more in-depth research as our results imply that only fostering self-enhancing attributions as well as self-efficacy do not seem to be enough to effectively reduce expression of the IP. Regarding negative features (Hypothesis 6b), we were able to reduce participants’ tendency to cover up errors and their fear of negative evaluation through the coaching intervention. This finding represents the successful progress in the coaching intervention when working on personal attitudes toward errors and evaluations specifically. Nevertheless, only the reduced fear of negative evaluation also had a significant effect on reducing expressions of the IP in our coaching participants. Thus, our findings indicate that interventions that are able to strengthen individuals to be less afraid of negative evaluations seem to be the most effective way for reducing IP fears. This is totally in line with original descriptions of the IP as impostors live in constant fear of being exposed as unintelligent or less competent (Clance, 1985; Harvey and Katz, 1985; Jöstl et al., 2012). If IP affected individuals are able to reduce their fear of being negatively judged by others, the IP will no longer be reinforced. If they are able to let go of their mask of perfection and also show their weaknesses, they will even be able to learn from mistakes. In turn, a mindset shift from an entity to a growth mindset will be further encouraged.

All in all, the interventions were created to focus on directly activating a growth mindset to reduce expressions of the IP. Indeed, the coaching intervention, where participants were required to actively engage in deep reflection and introspection, may have somehow fostered the development of a growth mindset and was explicitly effective in reducing IP scores. This offers further support for the findings of Spence et al. (2008): interventions that rely more on methods of facilitation and support than on education are able to achieve greater health behavior change. Generally speaking, the coaching intervention appeared to be an effective tool for improving the functioning of individuals in organizations and it facilitated individuals’ cognitive reframing of work experiences and attitudes, as suggested in previous research (Grant, 2001; Theeboom et al., 2014). Our analyses yielded several insights. There are a number of mechanisms that could be responsible for the effectiveness of interventions. Therefore, we considered two positive features, namely, self-enhancing attributions and self-efficacy, as well as two negative ones, namely, tendency to cover up errors and fear of negative evaluation, all of them central according to mindset theory (Dweck, 1986; Hong et al., 1999) and the IP. Guided by mindset theory, we made use of these indicators of a fixed and a growth mindset. A mindset shift could have been induced by the intervention if a change in the scores of the presumed indicators had occurred. Our findings showed that the coaching intervention was able to foster an increase in participants’ self-enhancing attributions and self-efficacy. This is in accord with past work (Green et al., 2006; Finn, 2007; Spence and Grant, 2007; Spence et al., 2008; Moen and Skaalvik, 2009). Moreover, we were able to reduce participants’ tendency to cover up errors and their fear of negative evaluation through the coaching intervention. Thus, an increase in positive as well as a decrease in negative features took place, and a shift from a fixed to a growth mindset in the coaching participants is indicated. Though it is important to notice that even if a mindset shift is indicated as all indicators were influenced as expected, only the fear of negative evaluation reduced IP fears effectively, what will be discussed later on.

Taken together, all our findings suggest that the coaching intervention can be seen as an effective way of reducing IP scores in young employees. Hence, we conclude that fostering a mindset shift in reducing the fear of negative evaluations by a coaching intervention is indeed an efficacious way to reduce expressions of the IP. The strength of this intervention is further supported by the finding that coaching participants reported significantly lower IP scores compared to participants who did not receive any intervention as well as those who received the training intervention. As we found an effect of time in addition to an effect of condition, we can further conclude that the intervention effect is not just sustainable but even increases over time. Although the effectiveness of coaching was suggested by previous research and demonstrated in other domains (Grant, 2001; Theeboom et al., 2014), our results provide the first evidence for such an effect regarding the IP.

### Limitations, Strengths, and Future Research

The investigation has several limitations, which also highlight potential future research opportunities. Even though we found mindset theory to be especially appropriate in this regard, we measured indicators of the mindsets instead of the perceived mindsets directly. We did so because we were building on previous work reporting relations between the IP and the indicators, such as self-efficacy beliefs (Jöstl et al., 2012; McDowell et al., 2015; Neureiter and Traut-Mattausch, 2017) or the fear and overgeneralization of failure (Clance and Imes, 1978; Chae et al., 1995; Thompson et al., 1998; Ross et al., 2001; Kumar and Jagacinski, 2006; Neureiter and Traut-Mattausch, 2016a). Hence, future studies could integrate a mindset measure such as the Implicit Theory of Intelligence Scale (Dweck et al., 2009) to make a mindset change even more explicit. Especially, as our parallel mediation analysis (including all indicators) showed that only the fear of negative evaluation is a significant mediating variable between the coaching intervention and the IP. Even if our coaching intervention effectively increased self-enhancing attributions and self-efficacy and decreased the tendency to cover up errors, we did not find any relation with the dependent variable IP scores. Regarding self-enhancing attributions, one explanation may be that in the present study we did not differentiate between attributional style in achievement situations and social situations, as previous research did: Brauer and Wolf (2016) found a relationship between attributional style and the IP in achievement situations, but no significant correlation in social situations. Building on this consideration, the missing link between self-enhancing attributions and reduced IP scores should be further researched using operationalizations that enable such differentiations. As already mentioned, our analysis further shows that a reduced fear of negative evaluation emerged as the only significant mediating variable between the coaching intervention and the reduced IP scores when all potential mediators were taken into account. Neither improved self-enhancing attributions and self-efficacy nor a reduced tendency to cover up errors could explain this relationship. The finding that fear of negative evaluation only partially mediates the treatment effect on the reduction of IP scores indicates that other non-measured mechanisms explain the efficacy of the intervention. Therefore, future research should additionally consider other potential mediating variables. Again building on mindset theory, the use of the Implicit Theory of Intelligence Scale (Dweck et al., 2009) may offer different insights. Furthermore, it could be especially interesting to include more variables that have already been shown to be related to the IP in the working context such as job satisfaction or perceived organizational support (McDowell et al., 2015; Vergauwe et al., 2015) and may be fostered through a coaching intervention. Another limitation concerns our measurement of the utility judgment. The items of the two-item scale by Kauffeld et al. (2009) showed only a small correlation which might indicate that the response behavior is inconsistent across the two items. Hence, we additionally calculated the analyses for each utility item separately, which revealed similar tendencies and still no significant results (*F*s < 1.60, *p*s > 0.210). Instead of using those two items, it would be more adequate to make use of more items in future research. Furthermore, the outcome measures were self-reported, and responses could be subject to a demand characteristic effect in which participants felt obliged to report less intense impostor fears. However, even if we cannot rule out such an effect, we assume it is less likely as it also did not appear in participants who received the training intervention. Another preventive factor for such an effect might be that data collection took place online and participants were informed that anonymity was assured. Another limitation concerns our sample composition. Despite being drawn from organizational settings, our participants were comparatively young employees (average age = 18.39). Moreover, the sample size was relatively small and the sample skews male (70/103). As these components significantly reduce the generalizability of our findings, a replication of our study using a bigger gender-balanced sample with more working experience would be revaluing.

Despite the above limitations, the present study has provided original data related to the use of interventions for reducing the IP and have extended the knowledge base on IP intervention research. Moreover, the current investigation has several strengths: first is the use of a randomized controlled outcome design. According to Cavanagh and Grant (2006), this is the qualitatively best design for quantitative outcome research and extremely difficult to achieve. In comparison, many studies have used single group or pre–post within-subject designs (e.g., Olivero et al., 1997; Grant, 2003; Jones et al., 2006; Orenstein, 2006). By using this research design, we showed that as few as nine units over three face-to-face coaching sessions can be effective in reducing IP scores, extending previous work finding effective short-term interventions (e.g., Burke and Linley, 2007; Grant, 2009). Future research should explore this issue and compare short-term to long-term interventions regarding the intensity of IP thoughts. Moreover, one should be explore at what point a more in-depth intervention such as psychotherapy is required.

Second, we investigated different forms of intervention, which enabled us to describe different effects. Our findings therefore provide some information on what intervention might be useful for the intended outcome, for instance, knowledge increase or behavior change. Third, in addition to showing an effect of the intervention on the outcome variable of interest, we investigated potential process variables, providing insight into what happens when interventions are applied. This should be of special interest to researchers exploring mindset theory as well as the practical impact of coaching in general. In particular, the impact of a coaching intervention could be researched regarding other aspects that were shown to be related to the IP as stated above. Coaching could be used to foster affected individuals’ organizational citizenship behavior and affective commitment as well as job satisfaction and perceived organizational support (Grubb and McDowell, 2012; McDowell et al., 2015; Vergauwe et al., 2015; Neureiter and Traut-Mattausch, 2016b). Moreover, coaching high-potential professionals who are concerned about the IP might increase their career planning, career exploration, career striving, career decision-making skills, and motivation to lead (Neureiter and Traut-Mattausch, 2016a, 2017).

### Practical Implications

A number of practical implications can be drawn from the study. Reflecting on all our findings encourages us to recommend a combination of intervention forms and settings. For instance, an intervention could start with a training session to convey content-related knowledge in an optimum way, followed by an individual coaching. Such a combination of settings could have synergetic effects, as in addition to receiving information, participants can see that others are affected by IP fears as well. Especially against the background that our supplementary analysis revealed that the fear of negative evaluation seems to be a very relevant mediating variable in the relation of an intervention and the IP. This takes into account early considerations that a group setting might be fertile ground for key experiences of recognition and esteem (Clance et al., 1995). Notable in this regard, our data suggest that a combination of settings is required, as the dyadic setting had especially strong effects.

Our findings highlight that career counselors need to help young employees to promote a growth mindset, especially by reducing their fear of negative evaluation, when they are confronted with the IP. They should assist young employees in managing their fear of failure better so that they become convinced that learning from failure fosters development. The findings clearly emphasize how important it is for career counselors to pay attention to young employees’ implicit theory. Coaching interventions could be adapted to foster growth beliefs about work; that is, they could convey information that targets the participants’ beliefs about work in addition to helping them internalize this information through experiences. Hence, we hope that coaching sessions like ours will encourage IP-affected individuals to feel as competent as they are and to believe in the growth of abilities even through learning from mistakes.

In addition, the comprehensive evaluation of the intervention effects allows us to draw conclusions about the acceptance of and need for IP-focused interventions in organizations. Employees’ immediate responses to the interventions constitute a measure of customer satisfaction, which often plays a role in management’s decision regarding the implementation and funding of future learning and development activities (Kirkpatrick, 1994, 1996). As suggested by satisfaction and utility ratings, the employees were satisfied with the interventions and considered them to be helpful and relevant to mastering their job role. Therefore, we are convinced that these kinds of interventions have the potential to benefit both the employee and the organization. Organizations should consider using external (or internal) counselors to provide individual coaching as a support mechanism in conjunction with employee development initiatives during employee establishment, thereby building a self-enhancing error culture at both an organizational and an individual level.

## Conclusion

The results presented in this paper show that different interventions can be used to work on the IP. Whereas a training intervention could be used to convey content-related knowledge, dyadic coaching sessions are especially effective in reducing IP scores. Such coaching is able to increase self-enhancing attributions and strong self-efficacy beliefs as well as decrease the tendency to cover up errors and fear of negative evaluation. These effects are desirable in general but also particularly considering the IP. This investigation is of high relevance for individuals as well as for organizations, bearing in mind that the IP has been shown to have a severe negative impact on high-potential employees.

## Data Availability Statement

The datasets generated and/or analyzed during the current study are available from the corresponding author on reasonable request.

## Ethics Statement

The study was approved by the ethics board of the University of Salzburg and carried out in strict accordance with their guidelines. Informed consent was obtained from all individual participants included in the study.

## Author Contributions

MZ, SJ, A-MW, and ET-M substantially contributed to the conception and the design of the work as well as in the analyses and interpretation of the data, worked for the final approval of the version that should be published, and are accountable for all aspects of the work in ensuring that questions related to the accuracy or integrity of any part of the work are appropriately investigated and resolved. MZ prepared the draft. SJ prepared parts of the draft. ET-M critically reviewed the manuscript and gave important intellectual input. A-MW entrusted with the development and evaluation of the coaching and training interventions and data collection.

## Conflict of Interest

The authors declare that the research was conducted in the absence of any commercial or financial relationships that could be construed as a potential conflict of interest.

## Footnotes

1 We excluded item 7 (“I was able to transfer the experience to my job”) from further analyses due to low internal consistency.

2 We changed the response scales of the ASF-E items from its original seven-point scale to a nine-point scale.

3 To test if goal attainment scores after the first session (T01) were comparable to T0 scores, we performed a mixed factorial ANOVA. There was a non-significant main effect of the measurement time points (T0 vs. end of first session T01), *F*(1,95) < 1, *p* = 0.368, η^2^ = 0.01, and a non-significant interaction effect between the measurement time points and the intervention condition, *F*(2,95) = 1.04, *p* = 0.359, η^2^ = 0.02, indicating that goal attainment scores at the end of the first session (T01) represent an equivalent measure of goal attainment scores at T0.

4 Effect sizes were calculated with Psychometrica.de calculation methods #4 and #5 (Lenhard and Lenhard, 2016).

## References

[B1] Bandura A. (1977). Self-efficacy: toward a unifying theory of behavioral change. *Psychol. Rev.* 84 191–215. 10.1037/0033-295x.84.2.191 847061

[B2] Bauer J. Festner D. Harteis C. Heid H. Gruber H. (2004). Fehlerorientierung im betrieblichen Arbeitsalltag. Ein Vergleich zwischen Führungskräften und Beschäftigten ohne Führungsfunktion [Error orientation in the workday. A comparison between executives and nonmanagement employees]. *Zeitschrift für Berufs-und Wirtschaftspädagogik* 100 65–82.

[B3] Bernard N. S. Dollinger S. J. Ramaniah N. V. (2002). Applying the big five personality factors to the impostor phenomenon. *J. Pers. Assess.* 78 321–333. 10.1207/s15327752jpa7802_07 12067196

[B4] Biberacher L. (2010). *Evaluation einer Coachingausbildung [Evaluation of a Coaching Training].* Unpublished diploma thesis, University of Regensburg, Regensburg.

[B5] Bond C. Seneque M. (2013). Conceptualizing coaching as an approach to management and organizational development. *J. Manag. Dev.* 32 57–72. 10.3109/0142159X.2012.643835 22455700

[B6] Brauer K. Proyer R. T. (2017). Are impostors playful? testing the association of adult playfulness with the impostor phenomenon. *Pers. Individ. Dif.* 116 57–62. 10.1016/j.paid.2017.04.029

[B7] Brauer K. Wolf A. (2016). Validation of the german-language clance impostor phenomenon scale (GCIPS). *Pers. Individ. Dif.* 102 153–158. 10.1016/j.paid.2016.06.071

[B8] Braumandl I. Amberger B. Falkenberg F. Kauffeld S. (2013). “Coachen mit Struktur – Konzeptcoaching. Ausbildung zum Coach für Karriere- und Lebensplanung (CoBeCe). Konzept, Theorie und Forschungsresultate. [Coaching with structure—Concept-coaching. Training to become a coach for career- and life-planning. Concept, theory, and research results],” in *Coaching-Praxisfelder. Forschung und Praxis im Dialog [Coaching fields of practice. Research and practice in dialog]*, eds Wegener R. Fritze A. Loebbert M. (Wiesbaden: Springer Verlag), 73–83.

[B9] Braumandl I. Dirscherl B. (2005). *Karrierecoachingkonzept für Studierende [Careercoachingconcept for students].* Regensburg: University of Regensburg.

[B10] Brinkmann R. D. (2008). *Techniken der Personalentwicklung: Trainings- und Seminarmethoden; mit Checklisten und Tabellen [Techniques for Further Education: Trainings and Seminar Tools; Checklists and Tables].* Frankfurt am Main: Verlag Recht und Wirtschaft GmbH.

[B11] Burke D. Linley P. A. (2007). Enhancing goal self-concordance through coaching. *Int. Coach. Psychol. Rev.* 2 62–69.

[B12] Burnette J. L. O’boyle E. H. Vanepps E. M. Pollack J. M. Finkel E. J. (2013). Mind-sets matter: a meta-analytic review of implicit theories and self-regulation. *Psychol. Bull.* 139 655–701. 10.1037/a0029531 22866678

[B13] Cavanagh M. Grant A. M. (2006). “Coaching psychology and the scientist–practitioner model,” in *The Modern Scientist–Practitioner: A Guide to Practice in Psychology*, eds Lane D. A. Corrie S. (New York, NY: Routledge), 146–157.

[B14] Chae J.-H. Piedmont R. L. Estadt B. K. Wicks R. J. (1995). Personological evaluation of clance’s impostor phenomenon scale in a Korean sample. *J. Pers. Assess.* 65 468–485. 10.1207/s15327752jpa6503_7 16367710

[B15] Chrisman S. M. Pieper W. Clance P. R. Holland C. Glickauf-Hughes C. (1995). Validation of the clance imposter phenomenon scale. *J. Pers. Assess.* 65 456–467. 10.1207/s15327752jpa6503_6 16367709

[B16] Clance P. R. (1985). *The Impostor Phenomenon: Overcoming the Fear That Haunts Your Success.* Atlanta, GA: Peachtree.

[B17] Clance P. R. (1986). *The Impostor Phenomenon: When Success Makes You Feel Like a Fake.* New York, NY: Bantam Books.

[B18] Clance P. R. Dingman D. Reviere S. L. Stober D. R. (1995). Impostor phenomenon in an interpersonal/social context: origins and treatment. *Women Ther.* 16 79–96. 10.1300/j015v16n04_07

[B19] Clance P. R. Imes S. A. (1978). The imposter phenomenon in high achieving women: dynamics and therapeutic intervention. *Psychother. Theory Res. Prac.* 15 241–247. 10.1037/h0086006

[B20] Clance P. R. O’Toole M. A. (1987). The imposter phenomenon: an internal barrier to empowerment and achievement. *Women Ther.* 6 51–64. 10.1300/j015v06n03_05

[B21] Claro S. Paunesku D. Dweck C. S. (2016). Growth mindset tempers the effects of poverty on academic achievement. *Proc. Natl. Acad. Sci. U.S.A.* 113 8664–8668. 10.1073/pnas.1608207113 27432947 PMC4978255

[B22] Cozzarelli C. Major B. (1990). Exploring the validity of the impostor phenomenon. *J. Soc. Clin. Psychol.* 9 401–417. 10.1521/jscp.1990.9.4.401

[B23] Doran G. T. (1981). There’s a S.M.A.R.T. way to write management’s goals and objectives. *Manag. Rev.* 70 35–36.

[B24] Döring S. A. (2009). *Philosophie der Gefühle [Philosophy of Emotions].* Frankfurt am Main: Suhrkamp.

[B25] Dweck C. S. (1986). Motivational processes affecting learning. *Am. Psychol.* 41 1040–1048. 10.1037/0003-066x.41.10.1040

[B26] Dweck C. S. Chiu C.-Y. Hong Y.-Y. (2009). Implicit theories and their role in judgments and reactions: a word from two perspectives. *Psychol. Inq.* 6 267–285. 10.1207/s15327965pli0604_1

[B27] Dweck C. S. Leggett E. L. (1988). A social-cognitive approach to motivation and personality. *Psychol. Rev.* 95 256–273. 10.1037/0033-295x.95.2.256

[B28] Finn F. A. (2007). *Leadership Development Through Executive Coaching: The Effects on Leaders’ Psychological States and Transformational Leadership Behaviour.* Unpublished doctoral thesis, Queensland University of Technology, Brisbane.

[B29] French B. F. Ullrich-French S. C. Follman D. (2008). The psychometric properties of the clance impostor scale. *Pers. Individ. Dif.* 44 1270–1278. 10.3389/fpsyg.2019.00671 31024375 PMC6463809

[B30] Fried-Buchalter S. (1997). Fear of success, fear of failure, and the imposter phenomenon among male and female marketing managers. *Sex Roles* 37 847–859. 10.1007/bf02936343

[B31] Gould S. (1979). Characteristics of career planners in upwardly mobile occupations. *Acad. Manag. J.* 22 539–550. 10.5465/255743

[B32] Grant A. M. (2001). *Towards a Psychology of Coaching: The Impact of Coaching on Metacognition, Mental Health and Goal Attainment.* Unpublished doctoral thesis, Macquarie University, Macquarie Park.

[B33] Grant A. M. (2003). The impact of life coaching on goal attainment, metacognition and mental health. *Soc. Behav. Pers. Int. J.* 31 253–263. 10.2224/sbp.2003.31.3.253

[B34] Grant A. M. (2008). Personal life coaching for coaches-in-training enhances goal attainment, insight and learning. *Coach. Int. J. Theory Res. Prac.* 1 54–70. 10.1080/17521880701878141

[B35] Grant A. M. (2009). *Workplace, Executive and Life Coaching: An Annotated Bibliography From the Behavioural Science and Business Literature Coaching Psychology Unit.* Sydney: Australia.

[B36] Grant A. M. (2012). An integrated model of goal-focused coaching: An evidence-based framework for teaching and practice. *Int. Coach. Psychol. Rev.* 7 146–165.

[B37] Grant A. M. Cavanagh M. (2004). Toward a profession of coaching: sixty-five years of progress and challenges for the future. *Int. J. Evid. Based Coach. Mentor.* 2 1–16.

[B38] Grant A. M. Curtayne L. Burton G. (2009). Executive coaching enhances goal attainment, resilience and workplace well-being: a randomised controlled study. *J. Posit. Psychol.* 4 396–407. 10.1080/17439760902992456

[B39] Grant A. M. Green L. S. Rynsaardt J. (2010a). Developmental coaching for high school teachers: executive coaching goes to school. *Consult. Psychol. J. Prac. Res.* 62 151–168. 10.1037/a0019212

[B40] Grant A. M. Passmore J. Cavanagh J. M. Parker M. H. (2010b). “The state of play in coaching today: a comprehensive review of the field,” in *International Review of Industrial and Organizational Psychology*, eds Hodgkinson G. P. Ford J. K. (New York, NY: Wiley-Blackwell), 125–167. 10.1002/9780470661628.ch4

[B41] Grant A. M. Stober D. (2006). *Evidence Based Coaching: Putting Best Practices to Work for Your Clients.* Hoboken, NJ: Wiley.

[B42] Green L. S. Grant A. M. Rynsaardt J. (2007). Evidence-based life coaching for senior high school students: building hardiness and hope. *Int. Coach. Psychol. Rev.* 2 24–32.

[B43] Green L. S. Oades L. G. Grant A. M. (2006). Cognitive-behavioral, solution-focused life coaching: enhancing goal striving, well-being, and hope. *J. Posit. Psychol.* 1 142–149. 10.1080/17439760600619849

[B44] Grubb W. L. McDowell W. C. (2012). The imposter phenomenon’s impact on citizenship behavior and employee commitment: flying under the radar. *J. Bus. Issues* 1 1–10.

[B45] Guay F. Vallerand R. J. Blanchard C. (2000). On the assessment of situational intrinsic and extrinsic motivation: the situational motivation scale (SIMS). *Motivat. Emot.* 24 175–213.

[B46] Harvey J. C. Katz C. (1985). *If I’m So Successful, Why Do I Feel Like a Fake? The Impostor Phenomenon.* New York, NY: St Martin’s Press.

[B47] Hayes A. F. (2013). *Introduction to Mediation, Moderation, and Conditional Process Analysis: A Regression-Based Approach.* New York, NY: Guilford Press.

[B48] Heider F. (1958). *The Psychology of Interpersonal Relations.* New York, NY: Wiley.

[B49] Henning K. Ey S. Shaw D. (1998). Perfectionism, the impostor phenomenon and psychological adjustment in medical, dental, nursing and pharmacy students. *Med. Educ.* 32 456–464. 10.1046/j.1365-2923.1998.00234.x 10211285

[B50] Hong Y.-Y. Chiu C.-Y. Dweck C. S. Lin D. M.-S. Wan W. (1999). Implicit theories, attributions, and coping: a meaning system approach. *J. Pers. Soc. Psychol.* 77 588–599. 10.1037/0022-3514.77.3.588

[B51] Hossiep R. Krügers C. (2012). *Das Bochumer Inventar zur berufsbezogenen Persönlichkeitsbeschreibung-6 Faktoren: BIP-6F [The Bochumer Inventory for Work Specific Personality Descriptions 6 Factors: BIP-6F].* Göttingen: Hogrefe.

[B52] Ianiro P. M. Lehmann-Willenbrock N. Kauffeld S. (2014). Coaches and clients in action: a sequential analysis of interpersonal coach and client behavior. *J. Bus. Psychol.* 30 435–456. 10.1007/s10869-014-9374-5

[B53] Jones R. A. Rafferty A. E. Griffin M. A. (2006). The executive coaching trend: towards more flexible executives. *Leader. Organiz. Dev. J.* 27 584–596. 10.1108/01437730610692434

[B54] Jones R. J. Woods S. A. Guillaume Y. R. F. (2016). The effectiveness of workplace coaching: a meta-analysis of learning and performance outcomes from coaching. *J. Occup. Organ. Psychol.* 89 249–277. 10.1111/joop.12119

[B55] Jöstl G. Bergsmann E. Lüftenegger M. Schober B. Spiel C. (2012). When will they blow my cover? *Zeitschrift für Psychologie* 220 109–120. 10.1027/2151-2604/a000102

[B56] Kauffeld S. Brennecke J. Strack M. (2009). “Erfolge sichtbar machen: Das Maßnahmen-Erfolgs-Inventar (MEI) zur Bewertung von Trainings [Making achievements visible: the action-success-inventory (MEI) for evaluating trainings],” in *Handbuch Kompetenzentwicklung [Handbook Competence Development]*, eds Kauffeld S. Grote S. Frieling E. (Stuttgart: Schäffer-Poeschel), 55–78.

[B57] Kemper C. J. Lutz J. Neuser J. (2012). Konstruktion und Validierung einer Kurzform der Skala Angst vor negativer Bewertung (SANB-5) [design and validation of a short form of the fear of negative rating scale (SANB-5)]. *Klinische Diagnostik und Evaluation* 4 343–360.

[B58] Kirkpatrick D. L. (1994). *Evaluating Training Programs: The Four Levels.* San Francisco, CA: Berrett-Koehler.

[B59] Kirkpatrick D. L. (1996). Great ideas revisited. *Train. Dev.* 50 54–59.

[B60] Klinkhammer M. Saul-Soprun G. (2009). Das “Hochstaplersyndrom” in der Wissenschaft [The impostor syndrome in science]. *Organisationsberatung, Supervis., Coach.* 16 165–182. 10.1007/s11613-009-0119-7

[B61] Kumar S. Jagacinski C. M. (2006). Imposters have goals too: the imposter phenomenon and its relationship to achievement goal theory. *Pers. Individ. Dif.* 40 147–157. 10.1016/j.paid.2005.05.014

[B62] Langford J. (1990). *The Need to Look Smart: The Impostor Phenomenon and Motivations for Learning.* Doctoral thesis, Georgia State University, Atlanta.

[B63] Legassie J. Zibrowski E. M. Goldszmidt M. A. (2008). Measuring resident well-being: impostorism and burnout syndrome in residency. *J. Gen. Intern. Med.* 23 1090–1094. 10.1007/s11606-008-0536-x 18612750 PMC2517942

[B64] Lenhard W. Lenhard A. (2016). *Berechnung von Effektstärken [Calculaton of effect sizes].* Dettelbach: Psychometrica Available online at: https://www.psychometrica.de/effektstaerke.html (accessed February 15, 2020).

[B65] Locke E. A. Latham G. P. (1990). *A Theory of Goal Setting & Task Performance.* Englewood Cliffs, NJ: Prentice Hall.

[B66] Losch S. Traut-Mattausch E. Mühlberger M. D. Jonas E. (2016). Comparing the effectiveness of individual coaching, self-coaching, and group training: how leadership makes the difference. *Fron. Psychol.* 7:629. 10.3389/fpsyg.2016.00629 27199857 PMC4853380

[B67] Mäthner E. Jansen A. Bachmann T. (2005). “Wirksamkeit und Wirkung von coaching [effectiveness and effects of coaching],” in *Handbuch Coaching [Handbook Coaching]*, ed. Rauen C. (Göttingen: Hogrefe), 55–76.

[B68] McDowell W. C. Grubb W. L. Geho P. R. (2015). The impact of self-efficacy and perceived organizational support on the imposter phenomenon. *Am. J. Manag.* 15 23–29.

[B69] McGregor L. N. Gee D. E. Posey K. E. (2008). I feel like a fraud and it depresses me: the relation between the imposter phenomenon and depression. *Soc. Behav. Pers. n Int. J.* 36 43–48. 10.2224/sbp.2008.36.1.43

[B70] Moen F. Skaalvik E. (2009). The effect from executive coaching on performance psychology. *Int. J. Evid. Based Coach. Mentor.* 7 31–49.

[B71] Muck A.-M. (2015). *No Faking Anymore! The Effectiveness of Coaching in the Reduction of Impostor Feelings in Comparison to Training and Waiting-List Control Group*. Unpublished master’s thesis, University of Salzburg, Austria.

[B72] Mueller C. M. Dweck C. S. (1998). Praise for intelligence can undermine children’s motivation and performance. *J. Pers. Soc. Psychol.* 75 33–52. 10.1037/0022-3514.75.1.33 9686450

[B73] Neureiter M. Traut-Mattausch E. (2016a). An inner barrier to career development: preconditions of the impostor phenomenon and consequences for career development. *Front. Psychol.* 7:48. 10.3389/fpsyg.2016.00048 26869957 PMC4740363

[B74] Neureiter M. Traut-Mattausch E. (2016b). Inspecting the dangers of feeling like a fake: an empirical investigation of the impostor phenomenon in the world of work. *Front. Psychol.* 7:1445. 10.3389/fpsyg.2016.01445 27729882 PMC5037221

[B75] Neureiter M. Traut-Mattausch E. (2017). Two sides of the career resources coin: career adaptability resources and the impostor phenomenon. *J. Vocat. Behav.* 98 56–69. 10.1016/j.jvb.2016.10.002

[B76] Olivero G. Bane K. D. Kopelman R. E. (1997). Executive coaching as a transfer of training tool: effects on productivity in a public agency. *Public Pers. Manag.* 26 461–469. 10.1177/009102609702600403

[B77] Orenstein R. L. (2006). Measuring executive coaching efficacy? the answer was right here all the time. *Consult. Psychol. J. Pract. Res.* 58 106–116. 10.3109/02699206.2014.927002

[B78] Oriel K. Plane M. B. Mundt M. (2004). Family medicine residents and the impostor phenomenon. *Fam. Med.* 36 248–252. 15057614

[B79] Poppe P. Stiensmeier-Pelster J. Pelster A. (2005). *Attributionsstilfragebogen für Erwachsene: ASF-E [Attributional Style Questionnaire for Adults ASF-E].* Göttingen: Hogrefe.

[B80] Rigotti T. Schyns B. Mohr G. (2008). A short version of the occupational self-efficacy scale: structural and construct validity across five countries. *J. Career Assess.* 16 238–255. 10.1177/1069072707305763

[B81] Ross S. R. Stewart J. Mugge M. Fultz B. (2001). The imposter phenomenon, achievement dispositions, and the five factor model. *Pers. Individ. Dif.* 31 1347–1355. 10.1016/s0191-8869(00)00228-2

[B82] Rowold J. (2004). *Karriereplanung: Deutsche Übersetzung des Fragebogens zur Karriereplanung von Gould, 1979 [Career Planning: German Translation of Gould’s 1979 Questionnaire on Career Planning].* Münster: University of Münster.

[B83] Rybowiak V. Garst H. Frese M. Batinic B. (1999). Error orientation questionnaire (EOQ): reliability, validity, and different language equivalence. *J. Organiz. Behav.* 20 527–547. 10.1002/(sici)1099-1379(199907)20:4<527::aid-job886>3.0.co;2-g

[B84] Salas E. Cannon-Bowers J. A. (2001). The science of training: a decade of progress. *Annu. Rev. Psychol.* 52 471–499. 10.1146/annurev.psych.52.1.471 11148314

[B85] Salas E. Tannenbaum S. I. Kraiger K. Smith-Jentsch K. A. (2012). The science of training and development in organizations: what matters in practice. *Psychol. Sci. Public Int.* 13 74–101. 10.1177/1529100612436661 26173283

[B86] Schroder H. S. Moran T. P. Donnellan M. B. Moser J. S. (2014). Mindset induction effects on cognitive control: a neurobehavioral investigation. *Biol. Psychol.* 103 27–37. 10.1016/j.biopsycho.2014.08.004 25149141

[B87] Schulz von Thun F. (2013). *Miteinander reden 3: Das “Innere Team” und situationsgerechte Kommunikation: Kommunikation, Person, Situation [Talking to one Another 3: The “Inner Team” and Situation-Based Communication: Communication, Person, Situation].* Reinbeck: Rowohlt.

[B88] September A. N. Mccarrey M. Baranowsky A. Parent C. Schindler D. (2001). The relation between well-being, impostor feelings, and gender role orientation among Canadian university students. *J. Soc. Psychol.* 141 218–232. 10.1080/00224540109600548 11372567

[B89] Sightler K. W. Wilson M. G. (2001). Correlates of the impostor phenomenon among undergraduate entrepreneurs. *Psychol. Rep.* 88 679–689. 10.2466/pr0.2001.88.3.679 11508002

[B90] Sonesh S. C. Coultas C. W. Lacerenza C. N. Marlow S. L. Benishek L. E. Salas E. (2015). The power of coaching: a meta-analytic investigation. *Coach. Int. J. Theory Res. Prac.* 8 73–95. 10.1080/17521882.2015.1071418

[B91] Spence G. B. Cavanagh M. J. Grant A. M. (2008). The integration of mindfulness training and health coaching: an exploratory study. *Coach. Int. J. Theory Res. Prac.* 1 145–163. 10.1080/17521880802328178

[B92] Spence G. B. Grant A. M. (2007). Professional and peer life coaching and the enhancement of goal striving and well-being: an exploratory study. *J. Posit. Psychol.* 2 185–194. 10.1080/17439760701228896

[B93] Theeboom T. Beersma B. Van Vianen A. E. M. (2014). Does coaching work? a meta-analysis on the effects of coaching on individual level outcomes in an organizational context. *J. Posit. Psychol.* 9 1–18. 10.1080/17439760.2013.837499

[B94] Thompson T. Davis H. Davidson J. (1998). Attributional and affective responses of impostors to academic success and failure outcomes. *Pers. Individ. Dif.* 25 381–396. 10.1016/s0191-8869(98)00065-8

[B95] Vergauwe J. Wille B. Feys M. De Fruyt F. Anseel F. (2015). Fear of being exposed: the trait-relatedness of the impostor phenomenon and its relevance in the work context. *J. Bus. Psychol.* 30 565–581. 10.1007/s10869-014-9382-5

[B96] Weidemann D. (2015). *Interkulturelles Lernen: Erfahrungen mit dem chinesischen’Gesicht’: Deutsche in Taiwan [Intercultural Learning: Experiences With the Chinese Face: Germans in Taiwan].* Bielefeld: Transcript.

[B97] Yeager D. S. Romero C. Paunesku D. Hulleman C. S. Schneider B. Hinojosa C. (2016). Using design thinking to improve psychological interventions: the case of the growth mindset during the transition to high school. *J. Educ. Psychol.* 108 374–391. 10.1037/edu0000098 27524832 PMC4981081

